# Hybrid Nanofluids—Next-Generation Fluids for Spray-Cooling-Based Thermal Management of High-Heat-Flux Devices

**DOI:** 10.3390/nano12030507

**Published:** 2022-02-01

**Authors:** Muhammad Asim, Farooq Riaz Siddiqui

**Affiliations:** 1 School of Professional Education & Executive Development, The Hong Kong Polytechnic University, Kowloon, Hong Kong 100077, China; 2 Department of Mechanical and Aerospace Engineering, The Hong Kong University of Science and Technology, Hong Kong 100077, China; frsiddiqui@connect.ust.hk

**Keywords:** hybrid nanofluids, high-heat-flux devices, electric vehicles, thermal management

## Abstract

In recent years, technical advancements in high-heat-flux devices (such as high power density and increased output performance) have led to immense heat dissipation levels that may not be addressed by traditional thermal fluids. High-heat-flux devices generally dissipate heat in a range of 100–1000 W/cm^2^ and are used in various applications, such as data centers, electric vehicles, microelectronics, X-ray machines, super-computers, avionics, rocket nozzles and laser diodes. Despite several benefits offered by efficient spray-cooling systems, such as uniform cooling, no hotspot formation, low thermal contact resistance and high heat transfer rates, they may not fully address heat dissipation challenges in modern high-heat-flux devices due to the limited cooling capacity of existing thermal fluids (such as water and dielectric fluids). Therefore, in this review, a detailed perspective is presented on fundamental hydrothermal properties, along with the heat and mass transfer characteristics of the next-generation thermal fluid, that is, the hybrid nanofluid. At the end of this review, the spray-cooling potential of the hybrid nanofluid for thermal management of high-heat-flux devices is presented.

## 1. Introduction

Hybrid nanofluids are a new class of heat transfer nanofluid engineered by dispersing two different types of nanoparticles in conventional heat transfer fluid (called the base fluid) [1,2,3]. Hybrid nanofluids offer enhanced heat transfer performance in thermal processes, exhibiting better thermophysical properties than conventional heat transfer fluids (oil, water and ethylene glycol) and mono nanofluids [4]. Recent research has indicated that hybrid nanofluids can replace mono nanofluids (comprising a single type of nanoparticles) as they provide better heat transfer enhancement in various thermal applications, such as automobile, electro-mechanical processes, manufacturing processes, HVAC and solar energy systems. The modern development in the field of engineering is increasing the demand of exceptionally featured compact devices with the best performance, accurate functioning and long lifespan. In order to meet high power density requirements in modern high-heat-flux devices, efficient heat transfer processes play a pivotal role. Therefore, in recent years, extensive research has been carried out on the thermal management of high-heat-flux devices to ensure their efficient cooling. However, despite promising heat transfer characteristics, there is limited research on the application of hybrid nanofluids to address heat dissipation issues in high-heat-flux devices. This is because hybrid nanofluid is still in its primitive stages of research where more emphasis has been on understanding its fundamental characteristics than its application in thermal systems.

Hybrid nanofluids are prepared by the dispersion of two different nanoparticles in a base fluid that provides synergistically enhanced thermal effects compared to traditional fluids and mono nanofluids [5]. The first study on hybrid nanofluids was carried out by Turcu et al. [6] on hybrid particulate fusion of nanocomposites, called multiwalled carbon nanotubes (MWCNTs), on Fe_2_O_3_ magnet nanoparticles and two different polypropylene-carbon nanotube (PPY-CNT) hybrids of nanocomposites. The thermal performance of hybrid nanofluid highly depends on the inter-particle compatibility of different nanoparticles used in it. For instance, the thermal conductivities of hybrid nanofluids (carbon nanotube copper and carbon nanotube gold) are lower compared to mono nanofluids due to inter-particle compatibility issues [7]. Several factors affecting the heat transfer enhancement of hybrid nanofluids have been identified, including nanoparticle synthesis, thermal conductivity, preparation methods, particle level, compatibility, shape and appropriate thermal network formation with fluid molecules [8,9,10,11,12]. Masuda et al. [13] dispersed micrometer-sized solid particles in single phase fluids and observed that the thermal conductivity of hybrid nanofluids enhanced but that there was sedimentation in the base fluid, which reduces the conductivity. In another study, Li and Xuan [14] observed an increase in the heat transfer by 60% for 2% concentration of Cu/H_2_O nanofluid in a tube at a Reynolds number of 25,000, and also developed an independent Nusselt number correlation for laminar and turbulent flow. Wen and Ding [15] conducted experimental analysis on Al_2_O_3_/H_2_O nanofluids in a tube under laminar flow and observed a 47% increase in the heat transfer at 1.6% volume fraction as compared to water as the base fluid. Duangthongsuk and Wongwises [16] found an increase in the heat transfer by 20% and 32% for 1.0% volume fraction of TiO_2_/H_2_O nanofluid flowing in a tube at Reynolds numbers of 3000–18,000, respectively, at a temperature of 38 °C. Sundar et al. [17] observed a 31% increase in the heat transfer with a pumping penalty of 10% for a 0.6% volume fraction of Fe_3_O_4_/H_2_O nanofluid in a tube at a Reynolds number of 22,000. Similarly, a lot of other researchers also observed heat transfer enhancement using hybrid nanofluids. The examples are as follows: Amrollahi et al. [18], Wang et al. [19] and Ding et al. [20] used carbon nanotubes nanofluids, Sajadi and Kazemi [21] used TiO_2_ nanofluids, Ghazvini et al. [22] used diamond/engine oil nanofluids, Ferrouillat et al. [23] used SiO_2_/water nanofluids and Guo et al. [24] obtained significant heat transfer rates by using Fe_2_O_3_/water nanofluids.

In addition to the ongoing research studies on existing nanofluids, it is important to discuss the potential of newly developed hybrid nanofluids. Mashhour et al. [25] studied the thermal performance and flow characteristics of a shell and tube heat exchanger with changing baffle angles using water and hybrid nanofluids at two different concentrations of 0.04% and 0.1% of GNP-Ag/water within the Reynolds number (Re) values ranging between 10,000 and 20,000. They found that, at a low Re number, the Nusselt number (Nu) corresponding to the baffle angle of 135° was very close to the recorded value at 180°. At Re = 20,000, the Nu number increased by 35% as compared to the reference case. M. Bahiraei et al. [26] evaluated the thermohydraulic attributes of a hybrid nanofluid containing graphene–silver nanoparticles in a microchannel heat sink equipped with ribs and secondary channels. Employing the hybrid nanofluid in the microchannel heat sink improves the heat sink performance significantly. They also found that by increasing either the concentration or the Re number, the temperature decreases and the flow experiences a greater pumping power at higher Re numbers and concentrations. Zhang et al. [27] worked on the preparation of a new hybrid nanofluid with excellent thermal conductivity and stability, named BiOIO_3_, using two-step synthesis. They applied five different dispersants to disperse the BiOIO_3_ nanoparticles. The best-performing nanofluids with a zeta potential value of 144.45 mV and particle size of 22.90 nm could be prepared with a polyvinylpyrrolidone (PVP) dispersant. The thermal conductivity value of BiOIO_3_ becomes larger with increasing concentration at 50 °C, having a peak value of 1.52 at a volume concentration of 0.134%. Said et al. [28] reviewed the understanding of different physical phenomena of modern hybrid nanofluids and their development. They investigated the research on the heat transport of nanofluids and the introduction of new 2D materials along with the potential applications of nanofluids.

In this review, hybrid nanofluids are discussed as potential next-generation thermal fluids for the thermal management of high-heat-flux devices (such as electric vehicle high-power electronics, high-power LEDs, laser diodes, etc.). Due to their synergistic thermal effects and overall hydrothermal properties, hybrid nanofluids may show enhanced heat transfer properties in phase-change processes (such as spray cooling) for the thermal management of high-heat-flux devices. Since modern high-heat-flux devices have heat dissipation flux in a range between 100 W/cm^2^ and 1000 W/cm^2^, hybrid nanofluids with their unusual thermal properties may address the thermal management issues in such devices. The main scope of this review paper is to address heat dissipation challenges in modern high-heat-flux devices with the application of next-generation hybrid nanofluids in phase-change processes, such as spray cooling. Phase-change processes offer much better heat transfer rates than single-phase cooling. However, existing fluids do not possess high heat transfer coefficients to address enhanced heat dissipation rates in high-heat-flux devices. Therefore, the main objective of this review paper is to highlight the importance of hybrid nanofluids as potential next-generation thermal fluids for high-heat-flux cooling applications. Moreover, this paper also addresses the research gaps, such as using hybrid nanofluids in phase-change processes with high heat transfer rates (such as spray cooling) to address thermal issues in state-of-the-art high-heat-flux devices.

## 2. Heat Dissipation Issues in High-Heat-Flux Devices

The development of the electronic industry towards miniaturization, high power density and 3D heterogenous integration demands effective thermal management solutions to improve the lifespan and reliability of the high-heat-flux devices. Mahajan et al. [29] reported that the power of computers doubled every 36 months and that the heat flux in large-scale electronic equipment reached up to 10^3^–10^4^ W/cm^2^ [30,31]. Such a high heat flux may tremendously increase the device operating temperature, posing risks for device safety and reliability [32]. About 55% of electronic failures are caused by the improper or poor thermal management of high-heat-flux devices [33]. According to the international technology roadmap for semiconductors, the generated heat from a single chip was enhanced from 330 W/cm^2^ in 2007 to 520 W/cm^2^ in 2011 [34]. Agostini et al. [35] suggested that it is difficult to manage a heat dissipation flux of 300 W/cm^2^ at 85 °C using existing cooling technologies. Different techniques have been investigated for the efficient cooling and enhanced thermal management of high-heat-flux devices. However, these techniques were insufficient to meet the high cooling demand of high-heat-flux devices [36,37].

**Figure 1:**
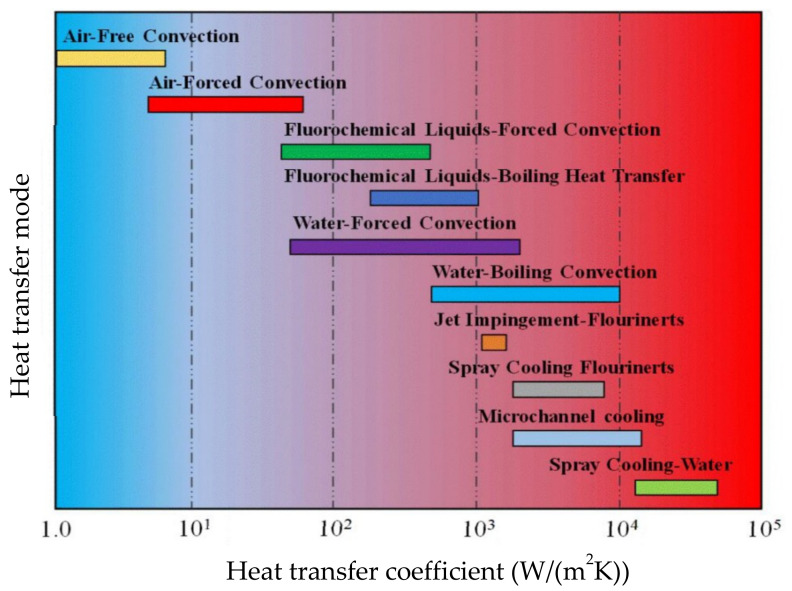
The cooling capacity of various cooling technologies. “Reprinted with permission from ref [48]. Copyright 2015 Elsevier”.

Presently, the main heat dissipation methods in high-heat-flux devices include natural and forced air cooling [38,39], fluorochemical liquid–forced convection and fluorochemical liquid–boiling heat transfer [40], forced water convective cooling [41], water boiling cooling [42], jet impingement [43,44,45,46], microchannel cooling [47,48,49,50] and spray cooling [51,52]. Figure 1 shows that air cooling cannot remove a heat dissipation flux above 100 W/cm^2^ and therefore cannot meet the heat dissipation requirement of the high-heat-flux devices. Additionally, the heat dissipation capability of conventional water cooling and heat pipes is also limited. Heat transfer mode in Figure 1 refers to the heat transfer mechanism that comprises both the cooling technology (such as pool boiling, jet impingement, microchannel, spray cooling, etc.) as well as the coolant (such as water, refrigerant, dielectric fluids, etc.) used in a cooling process. It is demonstrated that the air cooling mode of heat transfer is least effective among the existing cooling technologies. This is because air has a low heat transfer coefficient and high thermal contact resistance, which makes it an inefficient cooling medium for thermal applications. Figure 1 further illustrates that forced convection gives better heat transfer rates than free convection. Forced convection is followed by a boiling heat transfer mode for exhibiting a better heat transfer coefficient due to the phase-change process involved in it. The phase-change process involves latent heat energy with much higher heat transfer rates as compared to a single-phase heat transfer process utilizing sensible heat energy. However, the heat transfer coefficient in the boiling heat transfer mode is still less than the jet impingement and microchannel cooling processes due to the relatively high thermal contact resistance. The heat transfer coefficient in microchannel cooling is even higher than jet impingement due to micro-scaled channel dimensions offering an enhanced effective heat exchange area. Among all the heat transfer modes presented in Figure 1, spray cooling gives the highest heat transfer coefficient due to uniform surface cooling, high droplet area to volume ratio and low thermal contact resistance. Due to high heat transfer rates obtained from the spray-cooling process, this paper provides an extensive review on both hybrid nanofluids and spray-cooling technology with a recommendation to use hybrid nanofluids in spray-cooling processes to address heat dissipation issues in high-heat-flux devices. Therefore, advanced cooling methods are urgently needed for the effective cooling of high-heat-flux devices.

Heat transfer fluids commonly used for thermal management of high-heat-flux devices have poor thermophysical properties (as shown in Table 1) [53,54,55,56], making them incapable of addressing heat dissipation issues in high-heat-flux devices. Additionally, some dielectric coolants, for instance, Fluorinerts (FC-72, FC-84 and FC-87) and Performance Fluids (PF-5050, PF-5052, PF-5060 and PF-5070), have high global warming potential (GWP), making them inappropriate for high-heat-flux device cooling [54]. Furthermore, the cooling performance of water and traditional heat transfer fluids is much lower than the heat dissipation flux of some high-heat-flux devices, such as high-power electronics in electric vehicles. Researchers used water and dielectric fluids in microchannel heat sink, heat pipe, jet impingement and spray-cooling applications to cool high-power electronics in electric vehicles and reported heat flux removal in a range of 100−312 W/cm^2^ [54,57,58,59,60]. This is much below the required peak heat dissipation flux of 500 W/cm^2^ in current electric vehicles [61] and 1000 W/cm^2^ in future electric vehicles [62], thus presenting an urgent need for advanced thermal fluids, such as hybrid nanofluids.

## 3. Why Hybrid Nanofluid?

Nanofluid (also called mono nanofluid) is the colloidal suspension of very fine, nano-sized (below 100 nm) particles in a base fluid (such as water), which substantially improves its thermal properties and is widely reported by researchers [63,64,65]. The high thermal conductivity of mono nanofluids depends on various factors, such as the base fluid type, nanoparticle size, shape, type and its concentration [66,67,68]. The large area-to-volume ratio of highly conductive nanoparticles results in the higher thermal conductivity of mono nanofluids compared to their respective base fluids [69]. However, mono nanofluids do not possess overall hydrothermal properties, such as high stability and high thermal conductivity altogether. For instance, metal (such as copper) nanofluids show high thermal conductivity but poor dispersion stability. This is because metal nanoparticles are generally hydrophobic and do not form bonds with the surrounding water molecules. Such less stable nanofluids, when used in thermal applications, may result in sedimentation, clogging, fouling and system failures.

Although metal nanofluids can be stabilized using surfactants, their thermal properties are compromised, as surfactants cover the nanoparticle surfaces. Additionally, surfactants further increase the viscosity of metal nanofluids, resulting in high pumping power and large pressure losses. On the other hand, metal-oxide (such as Al_2_O_3_) nanofluids exhibit high dispersion stability, as metal-oxide nanoparticles are generally hydrophilic and can form bonds with the surrounding water molecules. However, metal-oxide nanofluids are thermally less conductive than metal nanofluids and are also not suitable for thermal systems due to their low heat rejection rates. Due to these reasons, mono nanofluids are not suitable for heat transfer applications, as they do not possess overall hydrothermal characteristics [70]. Recently, another class of nanofluid (known as the hybrid nanofluid) has been investigated, which has resulted in better overall hydrothermal properties than mono nanofluids and is prepared by dispersing two different nanoparticle types (metal, metal-oxide or non-metal) in the base fluid.

**Figure 2:**
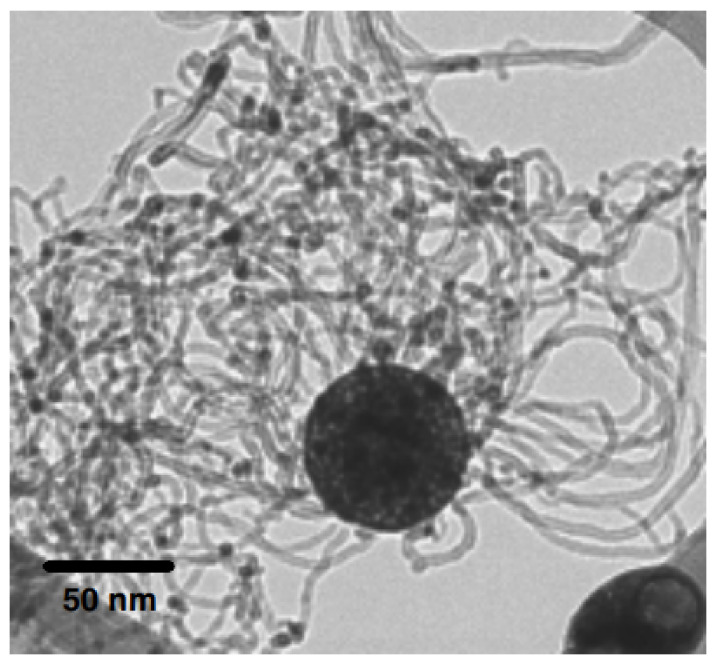
A TEM micrograph showing the thermal pathway of the (alumina/iron-oxide) sphere-CNT hybrid nanoparticle as a means of synergistic thermal effect in the hybrid nanofluid.

In addition to enhanced overall hydrothermal properties, the presence of two different nanoparticle types also has a synergistic thermal effect, thus making the hybrid nanofluid a highly conductive fluid, which is not the case with mono nanofluid. At even low particle concentrations, hybrid nanofluids are reported to exhibit higher thermal conductivity than mono nanofluids [71,72,73,74]. The synergistic thermal conductivity in the hybrid nanofluid is due to a thermal pathway created by one nanoparticle type with another nanoparticle type, thus reducing the overall thermal contact resistance between the nanoparticles and the surrounding molecules of the base fluid, shown in Figure 2 [75].

For this reason, the synergistic thermal effect in a hybrid nanofluid highly depends on the inter-particle compatibility. It is the synergistically advanced thermal properties and enhanced overall hydrothermal characteristics of the hybrid nanofluid that make it a potential candidate for the thermal management of high-heat-flux applications. Moreover, unlike most refrigerants, hybrid nanofluids (containing water as a base fluid) are environmentally friendly, as they do not exhibit global warming or ozone depletion issues. Additionally, as hybrid nanofluids can be used in closed loops in thermal applications, their chemical toxicity may not affect the environment.

### 3.1. Hybrid Nanofluid Synthesis

**Figure 3:**
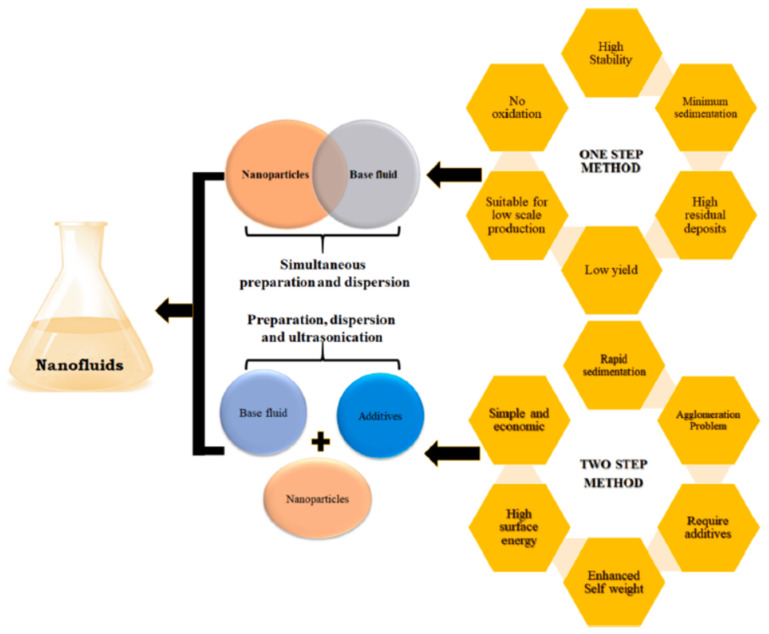
Nanofluids and their preparation. “Reprinted with permission from ref. [91]. Copyright 2021 Elsevier”.

The preparation and stability of the nanofluids are the initial requirements for the proper study and deploying them for appropriate applications. Several research papers [76,77,78] on the preparation of nanofluids, their stability and their thermophysical properties have already been published and reviewed in the past decade. Figure 3 shows the preparation methods of nanofluids: one-step and two-step methods with their advantages and disadvantages. The *single-step* preparation of the nanofluids involves the immediate preparation and dispersal of nanoparticles in the base fluid [79,80]. This method eliminated the drying and storage, resulting in less sedimentation, no oxidation and highly stable nanofluids. However, in this method of preparing nanofluids, the yield of the nanoparticles is very low, and this method is thus only suitable for small-scale production.

Unlike mono nanofluids, which are prepared by either a one-step (nanoparticles synthesized during nanofluid preparation) or a two-step method, hybrid nanofluids are generally synthesized using only a two-step method. This is because the one-step method involves the simultaneous production and dispersion of nanoparticles within the base fluid, which is difficult to implement in hybrid nanofluid synthesis. The two-step method involves the dispersion of already-prepared nanoparticles in the base fluid followed by mixing and ultra-sonication. In the hybrid nanofluid preparation, the two-step method can be further classified into two more types. In the first type, nanocomposite particles are first prepared and then dispersed in the base fluid. In the second type, two different nanoparticles are separately dispersed in the base fluid. In the first type, nanocomposite particles in the hybrid nanofluid are always prone to split into individual nanoparticles during ultra-sonication, which eventually becomes similar to dispersing different nanoparticles in the base fluid [8]. Additionally, the effect of the nanoparticle mixing ratio on the stability and hydrothermal characteristics of hybrid nanofluids is difficult to study using the first synthesis technique, since nanocomposite particle fabrication involves intricate chemical, physical or mechanical processes.

In a two-step method, the dispersed nanoparticles are mixed in the base fluid using a magnetic stirrer or a glass rod. Following mixing, the sample is ultra-sonicated using an ultra-sonication bath or a sonication probe [81,82,83,84]. Although an ultra-sonication probe results in better dispersion than an ultra-sonication bath due to direct probe immersion into the sample, ultra-fine particles detached from the probe during sonication may contaminate the hybrid nanofluid sample [69]. Moreover, ultra-sonication time, power and frequency are all important parameters that affect the hybrid nanofluid dispersion stability [85,86,87]. Furthermore, researchers suggested that high-pressure homogenizers can give even better nanoparticle dispersion in the base fluid compared to ultra-sonication [88,89,90]. Homogenizers involve impaction, cavitation and high shear stress inside their microchannel walls that break large aggregates into fine nanoparticles, thus resulting in a high dispersion stability [69].

The *two-step* method is an economic method, and, unlike the one-step method, the two-step method is used to produce the nanofluids in an extensive quantity [72]. Synthesized or commercially accessible nanoparticles are disseminated in a base fluid as a first step, and afterwards the second step involves ball millers, ultrasonics, homogenizers, etc. based on the specific requirements. This method is cost-effective and provides an improved performance of the nanofluids as compared to the one-step method [92,93,94].

The stability of the nanofluids is an important factor to be considered when selecting any nanofluid for a suitable application, as stability plays an important role in achieving enhanced thermal performance. Generally, nanoparticles show electrostatic and van der Waals force attractions. The grouping of the nanoparticles, due to the van der Waals interaction between nanoparticles, and sedimentation, due to the density difference between nanoparticles, causes instability. Enhancement in the stability of the nanoparticles can be achieved through pH control [95,96], ultrasonication [97,98], surfactant addition, surface modification techniques [99,100] and mechanisms [101]. Altering the pH value significantly changes the thermal conductivity of the nanofluids. Every nanofluid has a certain optimum pH for which maximum efficiency can be attained in the system.

The ultrasonication time and concentration of nanoparticles in the base fluid play a significant role in providing the uniform dispersion of the nanoparticles [98,102]. However, rapid sedimentation and accumulation is reported for usage beyond the optimum ultrasonication time. A summary of the various synthesis methods adopted by previous researchers is presented in Table 2.

### 3.2. Heat Transfer Characteristics of Hybrid Nanofluids

**Figure 4:**
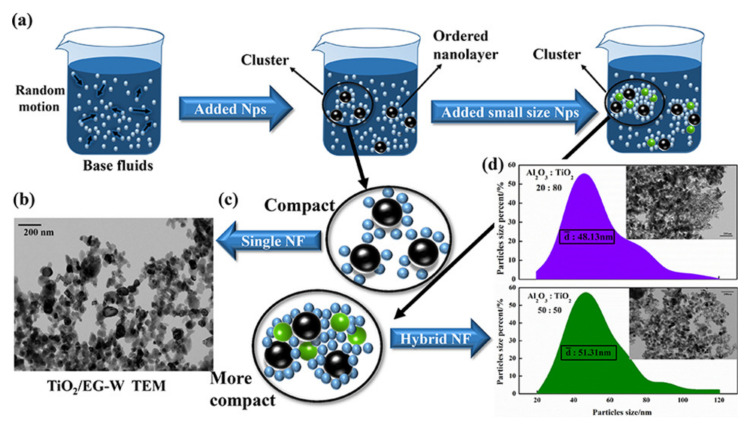
Demonstration of the synergistic thermal effect in hybrid nanofluids for the same volume fraction as mono nanofluids (**a**) Dispersed status (**b**) TEM of TiO_2_/EG-W nanofluid (**c**) inner structure of the cluster (**d**) TEM of Al_2_O_3_-TiO_2_/EG-W nanofluid. “Reprinted with permission from ref. [114]. Copyright 2020 Elsevier”.

The promising thermophysical properties of mono nanofluids show great potential in heat transfer enhancement in various applications. However, mono nanofluids (metallic, metal-oxide or non-metallic) do not exhibit overall hydrothermal properties (high stability and enhanced thermal conductivity). To overcome such shortcomings, hybrid nanofluids have been synthesized in recent years with synergistic thermal properties and enhanced heat transfer characteristics [113]. Heat transfer enhancement in hybrid nanofluids is mainly due to synergistic thermal effects, which is not the case with mono nanofluids for the same volume fraction of both mono nanofluids and hybrid nanofluids. Two different types of nanoparticles in hybrid nanofluids create a thermal network, thus reducing thermal contact resistance that cannot be achieved in mono nanofluids for the same volume fraction as used in hybrid nanofluids. When nanoparticles are dispersed in the fluid, the suspended nanoparticles become encapsulated by an orderly arrangement of surrounding fluid molecules called nanolayers, as shown in Figure 4. Theses nanolayers grow in size as more molecules surround the nanoparticle due to the Van der Waals force. These nanolayers act as a thermal bridge, thus reducing the thermal contact resistance between the nanoparticle and liquid molecules. Moreover, nanolayers possess intermediate thermal properties between nanoparticles and liquid molecules that help improve the overall thermal properties of nanofluids. However, the nanolayer thickness is greater in hybrid nanofluids compared to mono nanofluids for their same volume fractions, resulting in their synergistic thermal conductivity in hybrid nanofluids, as illustrated in Figure 4. This is because hybrid nanofluids comprise two different types of nanoparticles, resulting in a denser and more compact nanolayer around hybrid nanoparticles as compared to that obtained for the same volume fraction in mono nanofluids, as demonstrated in Figure 4.

Suresh et al. [110] carried out an investigation on the heat transfer and pressure drop characteristics of hybrid nanofluids. In his work, a fully developed laminar convective heat transfer and pressure drop characteristics through a uniformly heated tube using Al_2_O_3_-Cu/water were studied by developing an experimental test rig. The experimental setup consists of a calming section, test section, pump, cooling unit and fluid reservoir. The experimental results for laminar flow showed an enhancement of 13.56% in Nusselt number at a Reynolds number of 1730 when compared to water. There was an increase of 10.94% in Nusselt number for an Al_2_O_3_-Cu/water hybrid nanofluid when compared to pure water. Meanwhile, the enhancement obtained by 0.1% Al_2_O_3_/water nanofluid was 6.09% when compared to the pure water. This shows that introducing a small amount of copper nanoparticles in alumina matrix significantly enhances the Nusselt number. In another study conducted by Suresh et al. [115], they investigated the turbulent heat transfer and pressure drop characteristics of dilute Al_2_O_3_-Cu/water hybrid nanofluids and showed an average heat transfer enhancement of 8.02% compared to the pure water.

Pumping power is another important factor in cooling down the power electronic equipment using liquid-cooled heat sinks, because it is the only factor that determines the running cost of the cooling system [116]. Selvekumar and Suresh [117] worked on the effect of heat transfer and pressure drop characteristics using Al_2_O_3_-Cu/water hybrid nanofluids of 0.1% volume fraction in an electronic sink. The reported results proved that the pumping power increases with the increase in the volume flow rate of both deionized (DI) water and hybrid nanofluid. The pumping power required for the hybrid nanofluids was slightly higher than the DI water. An increase in the pumping power of 12.61% was observed when hybrid nanofluid was used as the coolant, which was less than the percentage rise in the convective heat transfer coefficient (24.35%). So, in light of the above discussion, they concluded that the hybrid nanofluids can be successfully used in the cooling of the electronic components.

In addition to this, there are several parameters that effect the thermal performance of the mono nanofluids and hybrid nanofluids. P.K Das [118] studied several factors affecting the thermal conductivity of the nanofluids and hybrid nanofluids. Some of them include nanoparticle type and size, pH, base fluids, solid volume fraction, temperature, sonication and surfactant. He also discussed synthesis, thermal conductivity characteristics and challenges in using the hybrid nanofluids. Sundar et al. [104] found that thermal conductivity augmentation in mono nanofluids and hybrid nanofluids is mainly attributed to the micro convection and particles’ Brownian motion in the base fluid. However, further enhancement in the thermal properties of hybrid nanofluids as compared to mono nanofluids is due to denser and more compact nanolayers resulting in synergistic thermal effects, as mentioned in the discussion of Figure 4. At a volume concentration of 0.3%, the thermal conductivity of MWCNT-Fe_3_O_4_-based nanofluid augmented by about 13.88% compared to that of base fluid at 20 °C. At T = 60 °C, it produces the higher thermal conductivity enhancement of 28.46%. Arvind et al. [119] observed that the graphene-MWCNT hybrid nanofluid showed an increase of 10.5% of thermal conductivity at a volume concentration of 0.04%, which was higher than pure graphene nanofluids. Similarly, Han et al. [75] measured the thermal conductivity of hybrid sphere/carbon nanotube-based polyalpha-olefin (PAO) oil nanofluids over a temperature range of 10–90 °C and observed a 21% enhancement in thermal conductivity in a PAO oil-based hybrid sphere/CNT nanofluids at a volume concentration of 0.2%, which is much higher as compared to the thermal conductivity of the nanofluids containing spherical nanoparticles of the same particle loading. G.M. Moldoveanu et al. [71] conducted an experimental investigation to study the thermal conductivity for two nanofluids (Al_2_O_3_–water and SiO_2_–water) and their hybrid (Al_2_O_3_–SiO_2_–water). The colloidal suspensions were analyzed at room temperature and at different temperatures (20–50 °C) and volume fractions (1–3%), respectively. The results showed an increase in thermal conductivity with an increase in the volume fraction and temperature. Moreover, the increase in the thermal conductivity of hybrid nanofluids depends on the volume fraction of both Al_2_O_3_ and SiO_2_ nanoparticles.

#### 3.2.1. Carbon Nanotubes (CNT) Based Hybrid Nanofluids

It is important to fully understand the heat transfer characteristics of hybrid nanofluids before using them in heat transfer applications. Since hybrid nanofluids have higher heat transfer enhancement as compared to the base fluids with a single nanoparticle, the researchers worked on the heat transfer of carbon nanotubes (CNT). Labib et al. [120] numerically investigated the heat transfer performance of a water and ethylene glycol (EG)-based CNT/water and mixture of Al_2_O_3_ into CNT using the two-phase mixture model and observed that the ethylene glycol-based nanofluids give better heat transfer rates as compared to water. Baby and Ramaprabhu [121] synthesized multi-walled carbon nanotubes (MWCNT), hydrogen exfoliated graphene (HEG) and Ag nanoparticles and prepared exfoliated graphene-based nanofluids. For a 0.005% volume concentration at a Reynolds number of 250, an increase of 570% in convective heat transfer enhancement is observed.

In another study by Arvind and Ramaprabhu [119], the synthesis of graphene and graphene/multiwalled CNT composite material was carried out. They prepared water-based nanofluids and found a 193% heat transfer enhancement at Re = 2000 for 0.02% volume concentration and suggested that these nanofluids are beneficial for the thermal management of the high-heat-flux devices (electronic cooling). Takabi and Salehi [122] numerically calculated the laminar natural convection for Al_2_O_3_-Cu/H_2_O hybrid nanofluids in a sinusoidal corrugated enclosure using discrete heat source on the bottom wall. They observed higher heat transfer rates for hybrid nanofluids as compared to the nanofluids with the same volume concentration.

#### 3.2.2. Oxide-Based Hybrid Nanofluids

Similar to the CNT-based hybrid nanofluids, the relevant literature related to the heat transfer of oxide-based hybrid nanofluids has also been investigated by different researchers. Suresh et al. [115] used Al_2_O_3_-Cu/H_2_O hybrid nanofluid flow in a tube under turbulent flow conditions and observed heat transfer enhancement of 8.02% at a volume fraction of 0.1%. Han and Rhi [123] prepared the hybrid nanofluids using different volume concentrations of silver/Al_2_O_3_-H_2_O used as working fluids in a grooved heated pipe. They investigated the heat transfer coefficients in the heat transfer rate for a power range of 50–300 W with 50 W intervals, volume fractions of 0.005%, 0.05% and 0.1% and inclinations of 5°, 45° and 90° and at cooling water temperatures of 1 °C, 10 °C and 20 °C and obtained better thermal performance with hybrid nanofluids in a grooved heat pipe.

## 4. Dispersion Stability

As hybrid nanofluids are prepared using a two-step method, they generally have low dispersion stability. However, some hybrid nanofluids show good stability in water, as they contain hydrophilic and chemically inert nanoparticles (metal-oxide nanoparticles such as Al_2_O_3_, MgO, etc.) that do not need any external stabilization mechanism [9,70]. Hybrid nanofluids containing hydrophobic (non-metal nanoparticles such as CNT, GNP, etc.) or chemically reactive (metal nanoparticles such as Cu, Ag, Zn, etc.) nanoparticles exhibit poor dispersion in water and therefore need stabilization [96]. There are three main methods used to improve the hybrid nanofluid stability, i.e., steric stabilization, surface treatment and electrostatic stabilization [124,125,126,127]. In steric stabilization, the surfactants (surface-active agents) are added into the hybrid nanofluid mixture that cover the hybrid nanoparticle surfaces. The surfactant acts as a bridge between the hybrid nanoparticle and surrounding fluid molecules, thus improving the dispersion stability [128,129]. The surfactants can be broadly classified as anionic (negatively charged), cationic (positively charged) or non-ionic (neutral). Although surfactants improve the dispersion stability, they can also increase the hybrid nanofluid viscosity and reduce its thermal conductivity [130,131].

Another method is the surface treatment of hydrophobic nanoparticles in which the nanoparticle surface is chemically modified and functional hydrophilic groups are attached to its surface, which improves the dispersion stability [132,133]. The main benefit of the surface treatment method is that it does not increase the viscosity of the hybrid nanofluid. In the electrostatic stabilization technique, the pH of the hybrid nanofluid is maintained far from its isoelectric potential (IEP) that induces an electrical double layer around hybrid nanoparticles, thus exhibiting high dispersion stability. The IEP is the pH where there is no net charge on hybrid nanoparticle surfaces. At the IEP, the absence of electrostatic repulsive forces causes particles to quickly agglomerate, which results in low dispersion stability [9,69,134].

**Figure 5:**
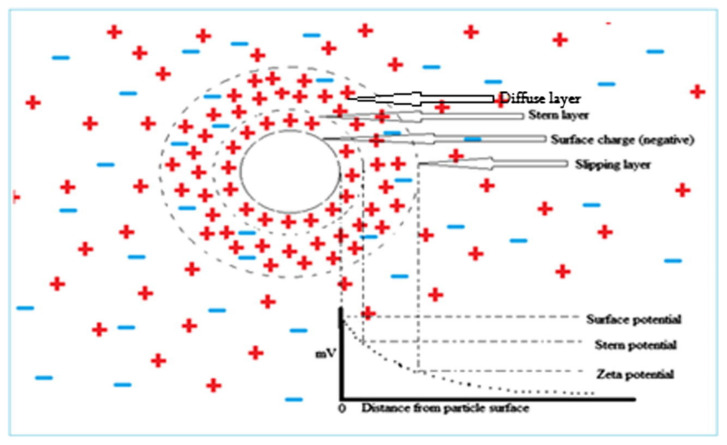
Different liquid layers around the suspended nanoparticle used in zeta potential measurement. “Reprinted with permission from ref. [69]. Copyright 2016 Elsevier”.

The hybrid nanofluid stability can be measured using various techniques, such as particle size analysis, zeta potential analysis, sedimentation analysis and UV-vis spectroscopy [135,136,137,138]. In particle size analysis, the effective diameter of suspended hybrid nanoparticles is measured over a period of time. The increase in particle size with time suggests agglomeration effects, indicating reduced stability. In zeta potential analysis, the net charge in the electrical double layer on suspended hybrid nanoparticles is measured (as illustrated in Figure 5), and therefore high zeta potential means high net charge on suspended particles and high dispersion stability due to large inter-particle repulsive forces.

**Figure 6:**
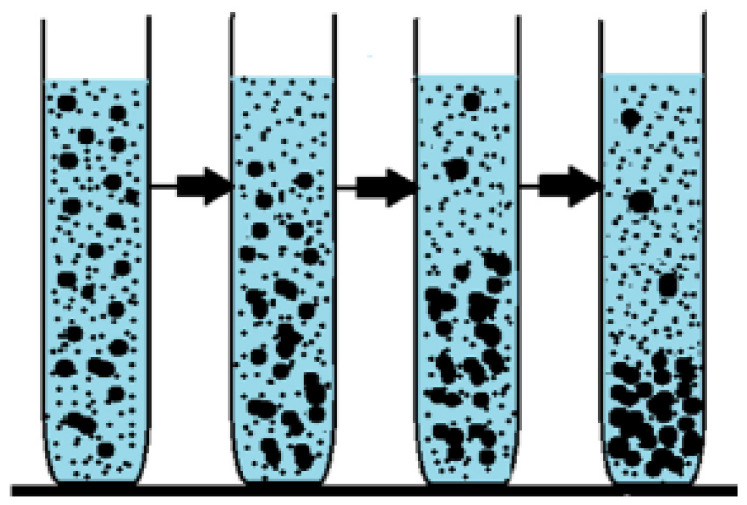
A schematic exhibiting the sedimentation process “Reprinted with permission from ref. [69]. Copyright 2016 Elsevier”.

Sedimentation analysis is a qualitative visual technique where sample images acquired over a certain period of time are analyzed to assess stability loss due to particle agglomeration, as demonstrated in Figure 6. UV-vis spectroscopy is based on the Beer–Lambert law, which states that light absorbance is linearly proportional to the concentration of colloidal particles in a suspension. As agglomerated particles sediment, less light is absorbed by the remaining suspended particles, and this information is used to determine the dispersion stability.

## 5. Thermophysical Properties

Hybrid nanofluids possess superior thermal properties compared to their respective base fluids and mono nanofluids. Thermal conductivity enhancement in a range of 16–32% using the hybrid nanofluid compared to the base fluid for particle concentrations up to a 2% volume fraction is reported in the literature [107]. Moreover, the hybrid nanofluid thermal conductivity considerably increases with increasing temperature and particle concentration [139,140,141]. Some other factors also affect the hybrid nanofluid thermal conductivity, such as the base fluid, surfactants, ultra-sonication time and nanoparticle type, size and shape [142]. Researchers suggested that thermal conductivity enhancement in hybrid nanofluids is mainly due to the Brownian motion and interfacial nano-layer surrounding the suspended hybrid nanoparticles in the base fluid. The Brownian motion of suspended hybrid nanoparticles generates micro-convection effects that increase the hybrid nanofluid thermal conductivity. On the other hand, the interfacial nano-layer comprises the liquid molecules at the solid–liquid interface that acts as a thermal bridge between the suspended nanoparticle and surrounding base fluid [143,144].

The hybrid nanofluid density depends on the density of the base fluid and respective densities and volume fractions of dispersed hybrid nanoparticles in the base fluid [94]. Similarly, the specific heat capacity of the hybrid nanofluid depends on the specific heat capacity and density of the base fluid and respective specific heat capacity, density and volume fraction of dispersed hybrid nanoparticles in the base fluid. Generally, the specific heat capacity of hybrid nanofluids decreases with increasing nanoparticle concentration and increases with increasing temperature [145,146]. The viscosity of hybrid nanofluids increases with increasing nanoparticle concentration and decreases with increasing temperature [104,146]. However, due to synergistic thermal effects and enhanced hydrothermal characteristics at even low particle loading, low concentrations of hybrid nanofluids can be used to reduce pumping losses and agglomeration issues in practical applications.

## 6. Mono Nanofluid and Hybrid Nanofluid Application in Phase-Change Cooling Processes

As the main scope of this review is to address the heat dissipation issues in modern high-heat-flux devices, it is important to discuss the application of existing mono nanofluids and next-generation hybrid nanofluids in efficient phase-change cooling processes, such as spray-cooling process. However, as spray cooling involves several droplets that may undergo evaporation or boiling processes, it is pertinent to first discuss the droplet evaporation behavior of mono and hybrid nanofluids in this review. Once the droplet evaporation and boiling behavior are well understood in this section, the next section (Section 6.2) will discuss the spray-cooling characteristics with emphasis on mono nanofluids and hybrid nanofluids.

### 6.1. Droplet Evaporation and Boiling

**Figure 7:**
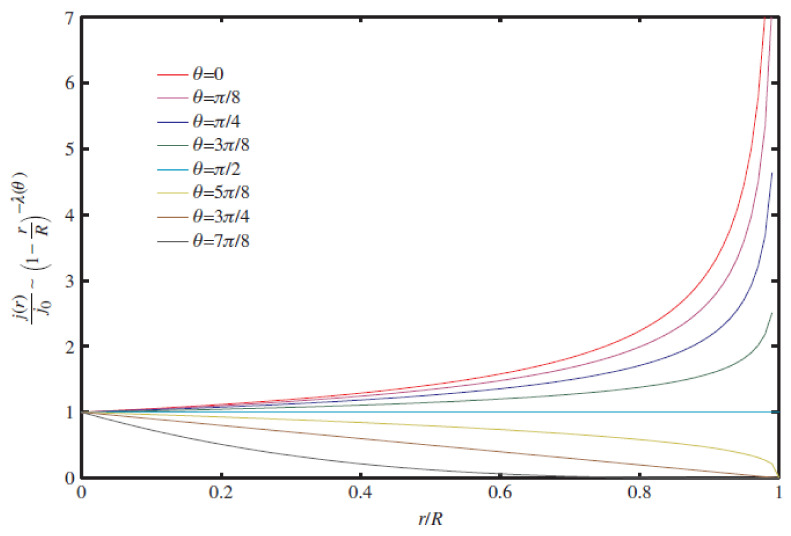
Dependence of sessile droplet evaporation flux along the droplet–air interface (j(r)) on its contact angle (θ). “Reprinted with permission from ref. [159]. Copyright 2021 Elsevier”.

**Figure 8:**
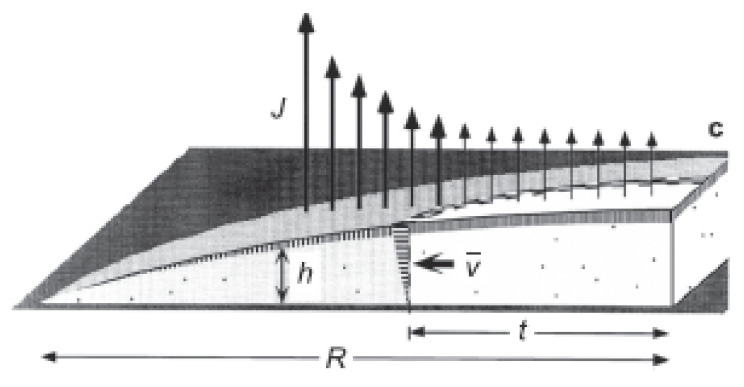
Increasing evaporation flux from the droplet apex to the three-phase contact line for contact angle θ < 90°. “Reprinted with permission from ref. [164]. Copyright 2015 AIP”.

**Figure 9:**
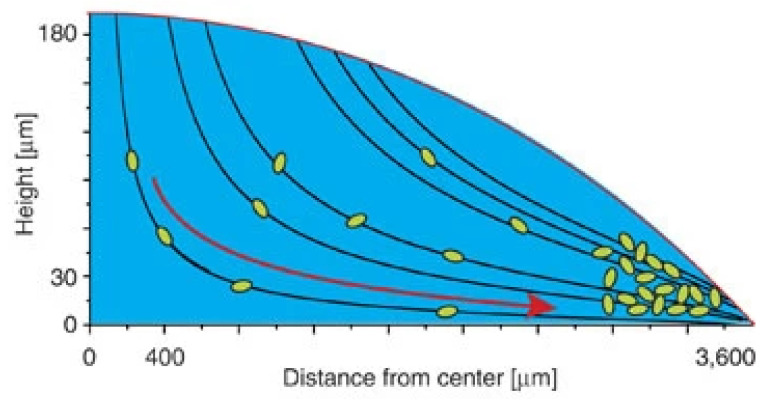
Outward movement of suspended particles inside the droplet due to non-uniform evaporation flux at the droplet–air interface. “Reprinted with permission from ref. [165]. Copyright 2013 Nature”.

In recent years, several hybrid nanofluids have been investigated for their enhanced thermophysical properties and improved dispersion stability. However, the hybrid nanofluid application in droplet-based cooling (such as spray cooling) remains an unexplored area to date. On the other hand, the droplet evaporation of mono nanofluids has been widely investigated. This may be because hybrid nanofluid research only recently started being comparable to nearly three decades of research on mono nanofluids. Many researchers investigated different residue patterns obtained from sessile nanofluid droplet evaporation over unheated surfaces. The droplet evaporation on unheated surfaces is generally treated purely as a diffusion process with negligible convection effects. During the droplet evaporation, the main mechanism controlling the liquid flow is of primary importance, as it determines the particle movement and the final deposit profiles. The capillary flow and the Marangoni flow are two important and major types of flow regimes frequently observed in evaporating sessile droplets. However, in many applications, a uniform deposition is preferred instead of a coffee ring style, so the capillary flow needs to be suppressed or even eliminated. A Marangoni flow with a reverse direction might work. Marangoni convention is driven by an uneven distribution of the liquid–vapor interface. The non-uniform distribution of liquid–vapor surface tension can result from a temperature gradient [147]. For thermally induced Marangoni flow, the direction of the convection is found by the non-uniform temperature distributions at the sessile droplet, which arise from the non-uniformity of the evaporation rate along the droplet and heat transfer non-uniformity from the substrate. The balance between these two sources of temperature distribution determines the direction of the Marangoni flow [148]. Moreover, the evaporation flux at the droplet–air interface depends on the droplet contact angle, as illustrated in Figure 7 and Figure 8. For droplet contact angles below 90°, the evaporation occurs non-uniformly over the droplet surface with increasing evaporation flux from the droplet centerline towards the three-phase contact line [149,150], as demonstrated in Figure 7. Once a droplet is pinned on the solid surface, the surface tensions initiate a radially outward flow to replenish the evaporation liquid loss at the periphery, known as capillary flow, as shown in Figure 9 [151]. As a result, the particles present in the liquid are driven outward and adsorbed at the three-phase line. The formation of the coffee ring pattern from a pinned colloidal droplet is ascribed to the capillary flow. This results in an outward movement of suspended particles along with the fluid from the droplet centerline towards the edge to replenish the vacant space from the evaporated fluid near the droplet edge, as shown in Figure 8. Consequently, a ring-shaped residue pattern is obtained due to the non-uniform evaporation flux of nanofluid droplets [152].

**Figure 10:**
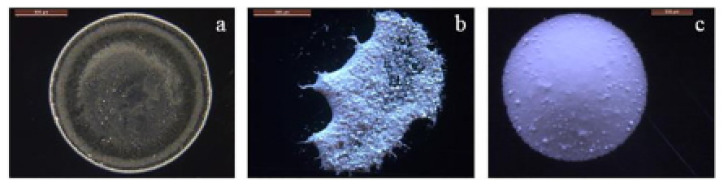
(**a**) Coffee-ring, (**b**) irregular and (**c**) uniform residue patterns obtained from Al_2_O_3_ nanofluid droplets for different nanoparticle sizes and concentrations. “Reprinted with permission from ref. [167]. Copyright 2017 Elsevier”.

However, other residue patterns (such as uniform and stick–slip patterns) can also be obtained depending on the base fluid, surfactant and nanoparticle type, size and concentration [153,154], as demonstrated in Figure 10. The nanofluid droplet generally undergoes a pinning effect (constant contact radius) for most of the droplet evaporation time. This is because the outwardly driven nanoparticles deposit near the droplet edge that pins the nanofluid droplet over the substrate. However, just before the evaporation ends, the nanofluid droplet shrinks over the substrate and enters the de-pinning mode (constant contact angle) [155,156]. The evaporation rate of the sessile nanofluid droplet over unheated surfaces mainly depends on the nanoparticle type, pinning effect, viscosity and presence of nanoparticles at the droplet–air interface [150,157,158].

Despite the potential benefits of hybrid nanofluids over mono nanofluids, no research effort was made to investigate the droplet evaporation performance of hybrid nanofluids over heated surfaces. However, the droplet evaporation of mono nanofluids on heated surfaces has been reported by a few researchers [160,161,162,163]. The mono nanofluid droplets exhibit higher evaporation rates, mainly due to their enhanced thermal conductivity, as compared to base fluid droplets over heated surfaces. As the particle concentration of evaporating nanofluid droplets increases with evaporation time, the effective thermal conductivity of the nanofluid droplets increases with evaporation time, resulting in enhanced nanofluid droplet evaporation rates as compared to base fluid droplets. Moreover, as particle migration occurs towards the droplet edge in evaporating nanofluid droplets due to internal convection effects, this results in an increased particle concentration near the droplet edge. Additionally, as the evaporation rate at the droplet edge is usually higher than the droplet surface, the increased particle concentration near the edge of the evaporating nanofluid droplet makes it a locally enhanced thermal conductive zone. This results in improved evaporation rates for nanofluid droplets as compared to their respective base fluid droplets.

Moreover, the enhanced evaporation rate of nanofluid droplets is also attributed to their smaller initial contact angles compared to those of base fluid droplets on heated surfaces. Al-Sharafi et al. [162] suggested that both Marangoni and buoyancy forces affect the internal flow field of the CNT nanofluid droplet over a hydrophobic surface. However, on other surfaces, they indicated that Marangoni forces have a dominating effect on the internal flow field as compared to natural convection [163].

Like droplet evaporation, the droplet boiling of hybrid nanofluids is an open yet demanding research area. A few researchers have investigated the droplet boiling mechanism in mono nanofluids. Okawa et al. [166] noticed that the titanium-dioxide (TiO_2_) nanofluid droplet shows high boiling heat transfer rates with a critical heat flux enhancement of 50% as compared to water droplets. They suggested that heat flux enhancement in nanofluid droplets may be due to nanoparticle deposition during the droplet nucleate boiling that alters the surface properties at the droplet–solid interface.

Duursma et al. [168] reported a 10% heat flux enhancement for a 0.1% volume fraction of aluminum-dimethyl sulfoxide (Al-DMSO) nanofluid droplets as compared to DMSO droplets. Kahani et al. [169] reported an up to 33% increase in cooling effectiveness for TiO_2_ nanofluid droplets as compared to water droplets over a heated silicon substrate. Paul et al. [170] investigated the Leidenfrost phenomenon for TiO_2_ nanofluid droplets and noticed that the evaporation time for concentrated TiO_2_ nanofluid droplets reduced about 10 times of that of water droplets in the film-boiling regime. They anticipated that vapor film underneath the TiO_2_ nanofluid droplet could not levitate it due to the high concentration of dense nanoparticles inside the TiO_2_ nanofluid droplet.

### 6.2. Spray Cooling

**Figure 11:**
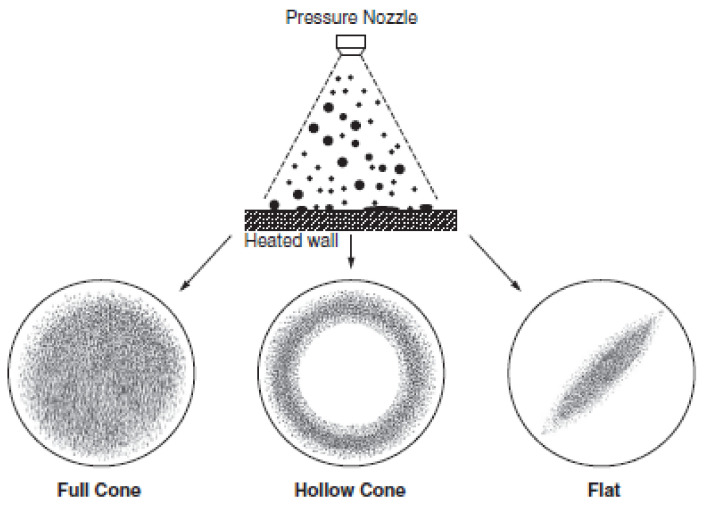
Different types of pressure spray nozzles. “Reprinted with permission from ref. [171]. Copyright 2017 Elsevier”.

As spray cooling involves numerous droplets of various sizes, the droplet evaporation and boiling behavior for mono nanofluids were discussed in the previous section. However, a lack of studies on hybrid nanofluid droplet phase-change processes serves as an opportunity for future researchers to address this promising research gap. Spray cooling has been widely investigated by researchers for its various parameters, such as number of nozzles, their type and orientation, fluid type, flow rate, film thickness and heater surface roughness. Pressure nozzles are generally preferred over air-assisted nozzles, as they do not need any secondary fluid stream for spray atomization [171]. Based on droplet distribution over the substrate, pressure nozzles are generally classified as full cone, hollow cone and flat type, as demonstrated in Figure 11. A few researchers reported that nozzle orientation has no impact on the spray-cooling performance, as gravity does not affect high-velocity spray droplets [172,173]. However, other researchers suggested that nozzle orientation affects the spray-cooling performance [174,175,176]. Mean droplet diameter, mean droplet velocity and volumetric flux are the main hydrodynamic parameters that influence the spray-cooling performance. Many heat transfer correlations based on Nusselt number and heat transfer coefficient were developed for single-phase spray cooling [177,178,179,180]. In the nucleate boiling regime, homogeneous nucleation, thin film evaporation and secondary nucleation were reported as the main mechanisms for phase-change heat transfer. In secondary nucleation, droplets induce bubble nucleation through vapor entrainment inside the thin film. Additionally, the impacted droplets increase nucleation frequency by breaking large bubbles into small-sized bubbles, thus resulting in secondary nucleation [181,182].

**Figure 12:**
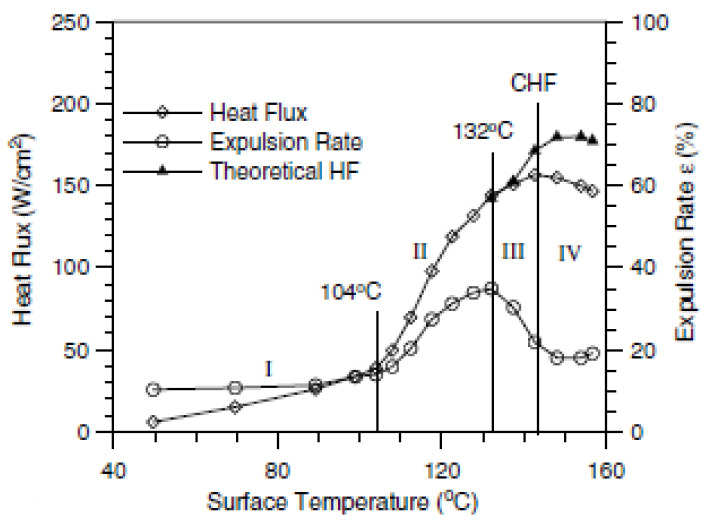
Variation in heat flux and expulsion rate for water spray cooling with a mass flux of 0.847 kg/m^2^s. “Reprinted with permission from ref. [183]. Copyright 2003 Elsevier”.

Jia and Qiu [183] suggested that spray-cooling heat transfer can be divided into four main regions using a parameter called expulsion ratio, as shown in Figure 12. They defined the expulsion ratio as the ratio of expelled mass flux to impacted mass flux over the heater surface. In region I, the heater surface temperature is below 100 °C, where droplets are mainly expelled due to splashing from spraying droplets and thin films on the heater surfaces. Region II initiates when the heater surface temperature is a little higher than 100 °C. In this region, the liquid film thickness decreases and it breaks into small fragments, resulting in an increasing expulsion rate. In region III, the expulsion rate decreases as the liquid film becomes even more thin and breaks into several droplets or disks. In this region, the nucleate boiling in thin film transforms into droplet evaporative cooling.

In region IV, the expulsion rate increases again due to the formation of vapor cushion at the droplet-heater interface. Several nucleate boiling heat flux correlations were developed based on heater surface temperatures, fluid thermophysical properties and spray hydrodynamic parameters [178,184,185,186]. Some contradictory findings were reported for the effect of heater surface roughness on spray-cooling performance. A few researchers concluded that smooth surfaces exhibit better heat transfer rates than rough heater surfaces [187,188], while others suggested that increasing surface roughness increases the spray-cooling performance [189,190,191,192,193]. Moreover, the critical heat flux (CHF) in spray cooling is mainly affected by hydrodynamic parameters, such as volumetric flux, mean droplet velocity and mean droplet diameter [194,195,196,197,198,199].

In addition to extensive research on spray-cooling performance using various fluids, the effect of additives (such as surfactants) on spray heat transfer enhancement was also investigated by researchers. They reported that adding surfactant up to a certain concentration increases the spray heat transfer rate; however, further increasing the concentration did not improve the spray-cooling performance [200,201,202].

**Figure 13:**
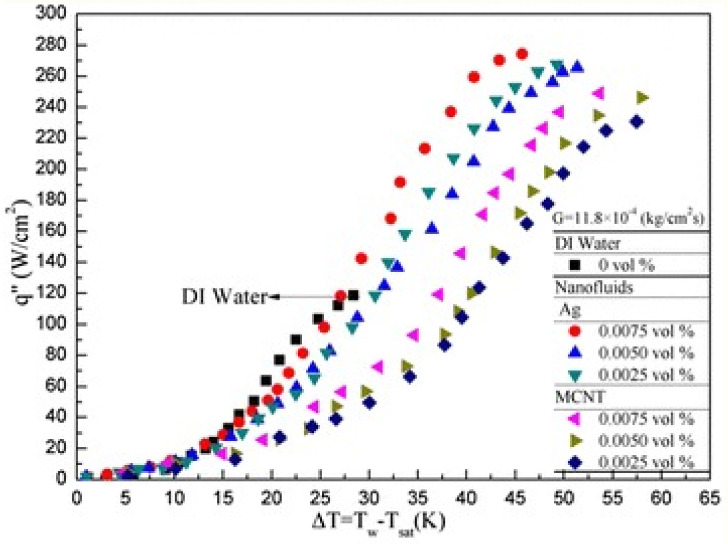
Comparison of nucleate boiling heat flux for water and mono nanofluids.”Reprinted with permission from ref [205]. Copyright 2015 Springer Open”.

Despite the high-heat-flux removal capability of hybrid nanofluid droplets compared to base fluid or mono nanofluid droplets, the spray-cooling potential of hybrid nanofluids has not been investigated to date. This research gap must be addressed in future studies to fully understand the spray-cooling potential of hybrid nanofluids. However, researchers reported contradictory findings on the heat transfer rates of mono nanofluid-based spray-cooling systems. Some researchers [203,204,205] indicated significant heat flux enhancements up to 2.4 times (as illustrated in Figure 13), while others suggested heat flux reduction using nanofluid spray cooling compared to base fluid spray cooling [168,206,207]. 

Moreover, the effect of nanoparticle loading on the spray-cooling performance of mono nanofluids is still unclear. Chang et al. [208] noticed substantial heat flux enhancement using a low particle loading of 0.001% volume fraction of an alumina nanofluid-based spray system. However, high particle loading in a range of 0.025–0.05% volume fraction deteriorated the spray-cooling heat flux. Tseng et al. [209] also indicated a decrease in heat transfer performance with increasing nanoparticle concentration in a range of a 1−40% mass fraction of titania nanofluid.

## 7. Conclusions

The present review reveals that, in recent years, the thermal management of high-heat-flux devices became a research focus due to increased power density, high output performance and dense packaging. This resulted in heat dissipation flux reaching unprecedented levels. For instance, heat flux in the high-power electronics of current electric vehicles (EVs) can reach up to 500 W/cm^2^ and it is anticipated to exceed 1000 W/cm^2^ in future EVs. Such a high heat flux may not be removed even by efficient cooling technologies (for instance, spray cooling) due to the limited heat removal capacity of existing thermal fluids, such as water and dielectric fluids. To address this issue, in this review, the cooling potential of the next-generation thermal fluid, called the hybrid nanofluid, based on a phase-change process, such as spray cooling, is discussed. Despite being an efficient cooling process, spray cooling may not address heat dissipation issues in high-heat-flux devices due to the low heat transfer coefficients and reduced heat transfer rates of existing thermal fluids. Even mono nanofluids are not ideal candidates for the thermal management of high-heat-flux devices for not possessing overall hydrothermal characteristics (i.e., high dispersion stability and enhanced thermal properties). On the other hand, hybrid nanofluids possess overall hydrothermal properties and synergistic thermal effects that make them suitable candidates for the thermal management of high-heat-flux devices. Hybrid nanofluids, when used in a spray-cooling process, may result in much higher heat flux removal rates compared to mono nanofluids or existing thermal fluids. The following are the main conclusions of this review:

- Hybrid nanofluids possess synergistic thermal effects and better overall hydrothermal characteristics compared to mono nanofluids.
- The thermal properties of hybrid nanofluids depend on the various parameters, such as type of base fluid and nanoparticles, nanoparticle concentration, size and shape.
- Hybrid nanofluids are generally prepared using a two-step method. The one-step method is not commonly used for hybrid nanofluid synthesis.
- Some hybrid nanofluids containing metal-oxide nanoparticles can be self-stabilized without any need for surfactants. However, other hybrid nanofluids containing non-metal nanoparticles (such as CNT and graphene) need surface treatment methods for stabilization.
- The stability of hybrid nanofluids is compromised at high particle concentrations due to agglomeration resulting in sedimentation.

## 8. Challenges and Future Work

### 8.1. Conventional Nanofluids

Although conventional nanofluids (or mono nanofluids) show enhanced heat transfer characteristics compared to existing thermal fluids, there are several challenges that need to be addressed for their application in high-heat-flux device cooling. The applicability of mono nanofluids is also limited by a lack of consensus on findings from various researchers, inadequate analysis of suspensions and a lack of standardized procedures for their preparation [210]. It is evident from the literature that one of the major challenges for mono nanofluids is their short-term dispersion stability [211,212], high pumping power and pressure drop [213], reduced thermal performance in turbulent flow [214], high viscosity [215], high cost [216] and limitation to mass production [217,218,219]. However, these challenges must be addressed in future research before conventional nanofluids can be considered for high-heat-flux device cooling application. Thermal characterization of nanofluids can help us to understand their performance mechanisms. Moreover, high temperatures can deteriorate the effect of dispersants due to effervescence issues [136,220]. As the durability of nanofluids is directly related to the properties of additives, future studies should prioritize the issues of additive selection and the performance of different types of surfactants for different nanofluids. Moreover, the effect of various ultrasonication parameters, such as ultrasonication time, power and frequency, on nanofluid stability must be considered in future research. Moreover, as the temperature of the suspension increases during ultrasonication processes, the nanofluid concentration may be changed due to fluid vaporization, thus affecting their properties. All these issues must be considered in future nanofluid research [221].

Moreover, there are no available concrete studies to justify the overall cost of different nanofluids. Traditional nanofluids are being replaced by other nanofluids mainly depending on their novel properties. Replacing conventional fluids with new nanofluids may give better thermal performance, but the overall cost of nanoparticles as well as the nanofluid preparation method is still high [136,222]. Therefore, the overall economics of different types of nanofluids should be investigated in future studies to increase the scope of their application in various fields.

### 8.2. Hybrid Nanofluids

**Figure 14:**
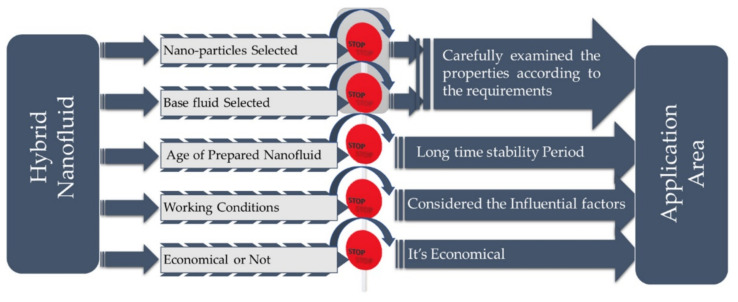
Challenges for hybrid nanofluid application in thermal systems. “Reprinted with permission from ref. [223]. Copyright 2019 Elsevier”.

Hybrid nanofluids have shown interesting characteristics in terms of heat transfer performance; however, there remain several challenges that need to be addressed in future research, such as the selection of appropriate composite nanoparticles and their preparation process, thermal conductivity models, stability and clogging issues. Additionally, there are some disagreements between the experimental data and theoretical models for hybrid nanofluids. Although much research has been performed to understand the hybrid nanofluid thermophysical properties, interparticle interactions and their effect on thermo-rheological characteristics need further research. Moreover, cost-effective methods must be developed for the preparation of hybrid nanofluids for their widescale application in thermal systems. Stability is another major obstacle for hybrid nanofluid application. To date, there is no detailed framework for the stability mechanisms of hybrid nanofluids. The thermophysical properties, stability and economic feasibility of hybrid nanofluids must be considered before their implementation in thermal applications, as illustrated in Figure 14.

Although the thermophysical properties of hybrid nanofluids have been investigated by the research community, their phase-change behavior is not fully understood to date. The synergistic thermal behavior and enhanced hydrothermal properties of hybrid nanofluids can be tapped in a spray-cooling process to address heat dissipation issues in high-heat-flux devices. Moreover, the spray-cooling potential of hybrid nanofluids should be investigated on high-heat-flux devices in future studies. Despite hybrid nanofluids possessing advanced thermal properties, their application in a spray-cooling process may result in a porous residue formation on a heated surface. Although hybrid nanofluid spray residues may enhance heat transfer rates due to capillary effects across residue micropores, they will need periodic cleaning to avoid fouling effects. Therefore, cleaning protocols must be developed in future studies for the periodic cleaning of deposited residues to avoid substrate fouling effects. Moreover, the cleaned residue comprising hybrid nanoparticles should be reused in order to retain the original concentration of hybrid nanofluids used in spray-cooling applications.

With all these issues addressed in future research, hybrid nanofluid spray cooling may emerge as a promising cooling technology to address heat dissipation issues in high-heat-flux devices.

## Figures and Tables

**Table 1:** Thermophysical property data of some fluids used in EV high-power electronics cooling [55,56].

Fluid	Saturation Pressure, P_sat_ (kPa)	Saturation Temperature, T_sat_ (°C)	Density, ρ (kg/m^3^)	Latent Heat of Vaporization, h_fg_ (kJ/kg)	Thermal Conductivity, k (W/m∙K)	Specific Heat Capacity, C_p_ (J/kg∙K)
**HFE-7100**	101.325	60.4	1372	112.1	0.062	1254
**FC-72**	101.325	56	1680	88	0.057	1100
**PF-5070**	101.325	80	1730	80	0.060	1050
**R-134a**	1700	60	1052	138.8	0.065	1669
**Water**	101.325	100	957.8	2257	0.68	4217

**Table 2:** Summary of various synthesis methods of hybrid nanoparticles [9].

Reference	Hybrid Nanoparticle	Base Fluid	Synthesis Method
Madhesh et al. [103]	Cu-TiO_2_	DI Water	Mechanical milling
Sundar et al. [104]	MWCNT-Fe_3_O_4_	Distilled water	In situ and chemical co-precipitation
Baby et al. [105]	MWNT-GO	DI Water	Catalytic chemical vapor deposition
Yarmand et al. [106]	GNP-Ag	Water	chemical vapor deposition
Batmunkh et al. [74]	Ag-TiO_2_	Water	Mechanical stirring
Abbasi et al. [107]	ϒ-Al_2_O_3_-MWCNT	Water	Solvothermal
Nine et al. [108]	Cu-Cu_2_O	Water	Wet ball milling
Chen et al. [109]	Ag-MWCNT	Water	Ball milling
Suresh et al. [110]	Al_2_O_3_-Cu	Water	Thermo chemical
Li et al. [111]	CNT-SiO_2_& CNT-SiO_2_-Ag	Water	Plasma treatment
Chen et al. [112]	MWCNT-Fe_3_O_4_	Water	Ball milling

## Nomenclature

*List of Abbreviations*		*List of Symbols*	
*Al-DMSO*	Aluminum dimethyl sulfoxide	*Ag*	Silver
*CHF*	Critical heat flux	*Al*	Aluminum
*CNT*	Carbon nanotubes	*Cu*	Copper
*DI* *EG*	Deionized Ethylene glycol	*Fe*	*Iron*
*EV’s*	Electric vehicles	*γ*	*gamma*
		*h*	*Free surface height*
		*H_2_O*	*Water*
*FC*	Fluorinets	*r*	*radius*
*GNP*	Graphene nanoparticles	*t*	*time*
*GWP*	Global warming potential	*Ti*	*Titanium*
*HEG*	Hydrogen exfoliated graphene		
*HFE*	Hydrofloroethers		
*HVAC*	Heat, ventilation and air-conditioning		
*IEP*	Isoelectric potential		
*LEDs*	Light emitting diodes		
*MWCNTs*	Multiwalled carbon nanotubes		
*MWNT*	Multiwalled nanotubes		
*PF*	Performance fluids		
*PAO*	Polyalpha-olefins		
*PPY-CNT*	Polypropylene carbon nanotubes		
*TEM*	Transmission electron microscopy		
*UV*	Ultraviolet		
